# Host Plant Effects on the Functional Response of *Scolothrips takahashii* to *Amphitetranychus viennensis*

**DOI:** 10.3390/insects17080840

**Published:** 2026-08-13

**Authors:** Pinhong Zhu, Ming Wei, Shien Pang, Zhenjie Hu, Junfeng Dong, Dingxu Li, Haibo Yang

**Affiliations:** 1 College of Horticulture and Plant Protection, Henan Key Laboratory of Insect Biology, Henan Provincial Engineering Technology Research Center of Green Plant Protection, Henan University of Science and Technology, Luoyang 471000, China; phzhu@haust.edu.cn (P.Z.); 19914950681@163.com (S.P.); zhenjiehu@haust.edu.cn (Z.H.); junfengdong@haust.edu.cn (J.D.); dingxli@haust.edu.cn (D.L.); 2 Henan Province Ryan Ping An Garden Plant Protection Co., Ltd., Zhengzhou 450001, China; 175122158@163.com

**Keywords:** *Scolothrips takahashii*, leaf trichome, functional response, hawthorn spider mite, host plant

## Abstract

The hawthorn spider mite, *Amphitetranychus viennensis*, can damage various Rosaceae fruit trees. When using predatory natural enemies for biological control, the effects of the physical characteristics of host plant leaves (such as trichomes) on the functional response of predatory natural enemies remain unclear. We studied the functional response of the predatory thrips, *Scolothrips takahashii*, to feeding on hawthorn spider mite eggs on five host plants (three apple cultivars: Golden Delicious, Fuji, and New Jonagold; peach; and plum). The results revealed that *S. takahashii* exhibited a typical type II functional response when feeding on mite eggs on Golden Delicious, peach, and plum, which had low trichome density. In contrast, it shifted to a type III functional response when feeding on mite eggs on the two apple cultivars, New Jonagold and Fuji, which had high trichome density. Additionally, the handling time of thrips in the type III response group was significantly longer than that in the type II response group, and the egg consumption at all prey densities was significantly lower than that in the type II response group. This study indicates that the shift in functional response of *S. takahashii* from type II to type III was associated with an increase in the density of trichomes on the host plant’s leaves, significantly reducing its predation efficiency. This finding reveals the mechanism by which plant physical traits indirectly regulate the predation strategies of natural enemies by influencing predation behavior, providing an important theoretical basis for evaluating the pest control potential of natural enemies and formulating variety layout in orchard habitats based on functional responses.

## Simple Summary

The hawthorn spider mite is a serious pest that damages various fruit trees. The predatory thrips, *Scolothrips takahashii*, can potentially control this pest; however, it remains unclear whether host plant traits affect its predation efficiency. We investigated the functional response of the thrips to mite eggs on five host plants: three cultivars of apple, peach, and plum. Additionally, we measured leaf trichome density. Our results showed that on apple cultivars with dense and long trichomes, the functional response of predators shifted from the typical type II to type III. This shift was associated with significantly longer handling times and lower egg consumption compared to plants with fewer trichomes. These findings indicate that dense leaf trichomes hinder thrips hunting and reduce their predation capacity, providing insights for selecting suitable fruit tree cultivars and optimizing biological control in orchards.

## 1. Introduction

The hawthorn spider mite, *Amphitetranychus viennensis* Zache, is a major economic pest of deciduous fruit trees worldwide, causing particularly severe damage to the Rosaceae family, such as apple, peach, plum, cherry, and apricot trees [1,2]. It often leads to premature leaf wilting, weakened tree vigor, and a significant decline in fruit quality [2]. For a long time, the control of this mite has relied heavily on chemical acaricides. However, due to its short generation cycle and high reproductive capacity, it has developed widespread resistance to various pesticides, exacerbating environmental risks such as pesticide residues and disruption of the ecological balance in fields [3,4,5]. Therefore, utilizing local natural enemies to implement integrated management strategies centered on biological control has become an urgent need for sustainable fruit tree production.

Functional responses describe changes in the feeding rates of individual predators in response to variations in prey density and can be used to assess the potential impact of predators on prey population dynamics [6,7]. The functional response of a predator is influenced not only by its interaction with prey but also by the physical and chemical properties of the habitat substrate [8]. Previous studies have shown that the morphological and biochemical characteristics of host plants can directly or indirectly influence the third trophic level of the food web [1,9], for example, by altering the search efficiency of predators and the number of prey captured [10,11]. Among these, the density and type of leaf trichomes are considered some of the most important plant traits influencing the activity of predatory natural enemies [12,13]. Since the search behavior of natural enemies is a key factor determining their functional response, it is reasonable to hypothesize that plant morphological traits (such as leaf trichomes) regulate the predation efficiency manifested in their functional response by influencing the search efficiency of natural enemies [14,15].

*Scolothrips takahashii* Priesner is one of the key predatory thrips species in East Asian orchard ecosystems and has significant potential for controlling various leaf mites, including the hawthorn spider mite [16,17,18]. Previous studies have shown that the functional response of *S. takahashii* to *A. viennensis* eggs typically follows the Holling-II pattern, meaning that predation rates increase hyperbolically as prey density rises, while the proportion of prey consumed continues to decline [19]. However, growing evidence suggests that host plants can profoundly influence the functional response parameters and even the response type of predators by altering the nutritional quality of prey or directly impeding the movement of natural enemies. For example, Merlin et al. (2022) found that when the predatory mite *Neoseiulus californicus* feeds on eggs of the two-spotted spider mite *Tetranychus urticae* on cotton plants, the functional response shifts from the typical type II to type III due to a significant reduction in the caloric content of the prey eggs [20]. Type III functional response exhibits an S-shaped curve, characterized by a low predation rate at low prey densities that increases as density rises. Theoretically, this has a density-dependent regulatory effect that is more conducive to the stability of population systems [6,21]. In addition, sublethal pesticide stress can induce a shift in the functional response type of natural enemies [22]. These studies suggest that the functional response type of predators may be an adaptive response to environmental stress, whether nutritional or chemical stress.

Currently, there is a lack of research on how different Rosaceae fruit tree species influence the functional responses of the *S. takahashii*—*A. viennensis* system. In particular, whether differences in the trichome characteristics of host plant leaves are sufficient to alter the functional response patterns of predators, and how such changes affect the pest control efficacy of natural enemies, remain urgent scientific questions. In this study, we selected three common apple cultivars (Golden Delicious, Fuji, and New Jonagold), as well as peach and plum, as host plants. We measured the density and length of trichomes on the undersides of leaves and evaluated the functional responses of female adult *S. takahashii* after feeding on *A. viennensis* eggs reared on the respective hosts. The aim is to elucidate the interaction mechanisms among the three trophic levels, plant, herbivore and predator, thereby providing a theoretical basis for the conservation and utilization of natural enemies in orchard habitats.

## 2. Materials and Methods

### 2.1. Mite and Thrips Colony

The colonies of *A. viennensis* and *S. takahashii* were both collected from an apple orchard in the suburbs of Luoyang City, Henan Province, northern China (112°22′ E, 34°37′ N). The mites were reared on detached apple leaves (*Malus pumila* Mill. cv Golden Delicious) placed upside down on a 5 mm layer of agar (1.5% *w*/*v*) in Petri dishes (150 mm diameter). To provide ventilation and prevent excessive humidity, the Petri dish lids were raised using 5 mm thick rubber sheets. The Petri dishes were placed in a climate chamber (RSH-250 II, Guangzhou Medical Device Factory, Guangzhou, China) under the following conditions: 25 ± 1 °C, 60 ± 10% RH, and a 16:8 h (L:D) photoperiod. Thrips pupae were placed in glass Petri dishes (100 mm in diameter and 20 mm in height) and allowed to emerge at 25 °C. Newly emerged adults were paired and introduced onto apple leaves infested with a specific number of spider mites. Each leaf was placed in a Petri dish (180 mm in diameter) as described above and stored in another climate chamber set to the same conditions. Adult thrips were transferred to newly infested apple leaves every 2 days to separate the eggs from the adults. The leaves and agar were replaced every 3 and 6 days, respectively. After a rearing period of 3 months, the colonies were used for subsequent experiments.

### 2.2. Host Plants

Female adult mites were selected from the aforementioned hawthorn spider mite colonies and transferred to detached leaves of the following five host plants for further cultivation: three apple cultivars (Golden Delicious, Fuji, and New Jonagold), peach (*Prunus persica* L. cv. Baifeng, a local cultivar), and plum (*Prunus domestica* L. cv. Blue Diamond). Prior to the experiment, the mites were reared on each host plant for at least three generations.

The number and length of trichomes (straight and hooked) on the undersides of fully expanded leaves from three apple cultivars (Golden Delicious, Fuji, and New Jonagold), peach, and plum were measured under a stereomicroscope (10× magnification). The number of trichomes within an area of 1 cm^2^ was counted, and the length of the trichomes was precisely measured after they were flattened using a microscope slide. From each host plant, ten plants with similar growth were randomly selected, and from each plant, take one functional leaf (the 3rd to 5th mature leaf counting downward from the tip of the branch) for trichome observation. A total of 50 leaves were analyzed from the aforementioned five host plants.

### 2.3. Functional Response

Leaf discs (30 mm in diameter) were cut from the leaves of various host plants after removing the midribs to serve as test arenas. Each disc was placed upside down on an agar pad (1.5% *w*/*v*), which was surrounded by water and positioned inside a multi-purpose insect rearing box (DZ-8003, Xi’an Dizhai Plant Protection Equipment Factory, Xi’an, China; 90 mm× 90 mm× 45 mm) to prevent predators from escaping. The rearing box was made of Perspex, featuring a square base (15 mm high), trapezoidal sides, and a circular top fitted with a lid. It was equipped with two 5-mm-diameter ventilation holes covered with fine nylon mesh. The box was placed inside a climate chamber, where the environmental conditions were identical to those described above.

The specific procedure is as follows: To obtain synchronized mite eggs, 10–30 *A. viennensis* females in peak oviposition were introduced onto leaf discs from different host plants. The mites were allowed to lay eggs for no more than 24 h, after which they were removed. Under a stereomicroscope, 10, 20, 30, 40, 50, and 60 eggs of *T. viennensis* were randomly selected using a brush, for a total of six density gradients. Subsequently, one *S. takahashii* female (4–6 days old) that had been starved for 12 h was placed onto each leaf disc. After 24 h, the thrips were removed, and the number of remaining eggs was inspected and recorded. If the predator escaped, the data were discarded. No prey was replenished during the experiment. For each host plant, there were 15 to 19 valid replicates for each prey density.

### 2.4. Statistical Analysis

#### 2.4.1. Trichome and Predation

Comparisons of trichome number and length among different plant species were performed using one-way analysis of variance (ANOVA) and Tukey’s HSD post-hoc test. When comparing predation on different host plants, since the data under egg densities of 10–30 eggs did not meet the assumptions of normality (Shapiro–Wilk test) and homogeneity of variances (Levene’s test), the Kruskal–Wallis test and Dunn’s post-hoc test were used. In contrast, for egg densities of 40–60 eggs, the predation data were analyzed using one-way ANOVA and Tukey’s HSD post-hoc test. In addition, we used the ‘glm’ function in R 4.6.0 to build a generalized linear model to assess the effects of density, host type, and their interaction on the predation rate (a binomial response variable).

#### 2.4.2. Functional Response

Functional response data were analyzed using the *Frair* package in R v.4.6.0 [23,24]. The analysis procedure consisted of three steps, model selection, model fitting, and comparison of the fitted results and coefficients, all conducted in accordance with the description provided by Tonga et al. (2025) [23]. Specifically, generalized linear models were used to distinguish between type II and type III functional responses. If the coefficient of the linear density term (first-order term) was significantly negative, the response was classified as type II; if the coefficient of the first-order term was significantly positive and the coefficient of the second-order term (density^2^) was significantly negative, the response was classified as type III. The analysis accounted for prey consumption and the absence of prey supplementation during the experiment. The predator’s type II functional response was then fitted using the “Lambert-W” function proposed by Rogers (1972) [25]:

*N*_e_ = *N*_0_[1 − *exp*(*a*(*N*_e_*h* − *T*))].

The type III functional response uses the equation proposed by Real (1977) [21]:

$$N_{e}=N_{0}[1-\exp\left(bN_{0}^{q}(N_{e}h-T)\right)],$$

where *N*_e_ represents the number of *A. viennensis* eggs consumed, *N*_0_ represents the initial supply of mite eggs, *a* is the attack rate, *h* is the handling time, *T* represents the total available time, *b* is the capture coefficient, and *q* is the scaling exponent.

Using a nonparametric bootstrap method, 95% confidence intervals were constructed based on the initial maximum likelihood estimates of *a* and *h* near the mean of the functional response curves under each treatment condition. Since *a* and *h* represent different biological processes, direct comparisons between type II and type III functional responses are not appropriate [26]. For host–prey systems, the “difference method” described by Juliano (2001) was employed to compare the differences in functional response component parameters (*a* and *h*) by performing 95% confidence intervals on the optimized coefficients using the ‘frair::frair_boot’ function [27]. The significance level for all statistical tests was set at *p* = 0.05.

## 3. Results

### 3.1. Trichome Density and Length

There were significant differences in trichome density and length among different host plants (Table 1; density: *F* = 54.548, *df* = 4, 45, *p* < 0.001; length: *F* = 9.778, *df* = 4, 45, *p* < 0.001). Among them, the New Jonagold apple cultivar had the highest trichome density, followed by Fuji; there were no significant differences in density among Golden Delicious, peaches, and plums, but all were significantly lower than those of New Jonagold and Fuji. The trend in trichome length was similar to that of density: New Jonagold trichomes were not significantly longer than those of Fuji, but were significantly longer than those of the other three host plants.

### 3.2. Effect of Host Plant on the Functional Response of Scolothrips takahashii

**Figure 1:**
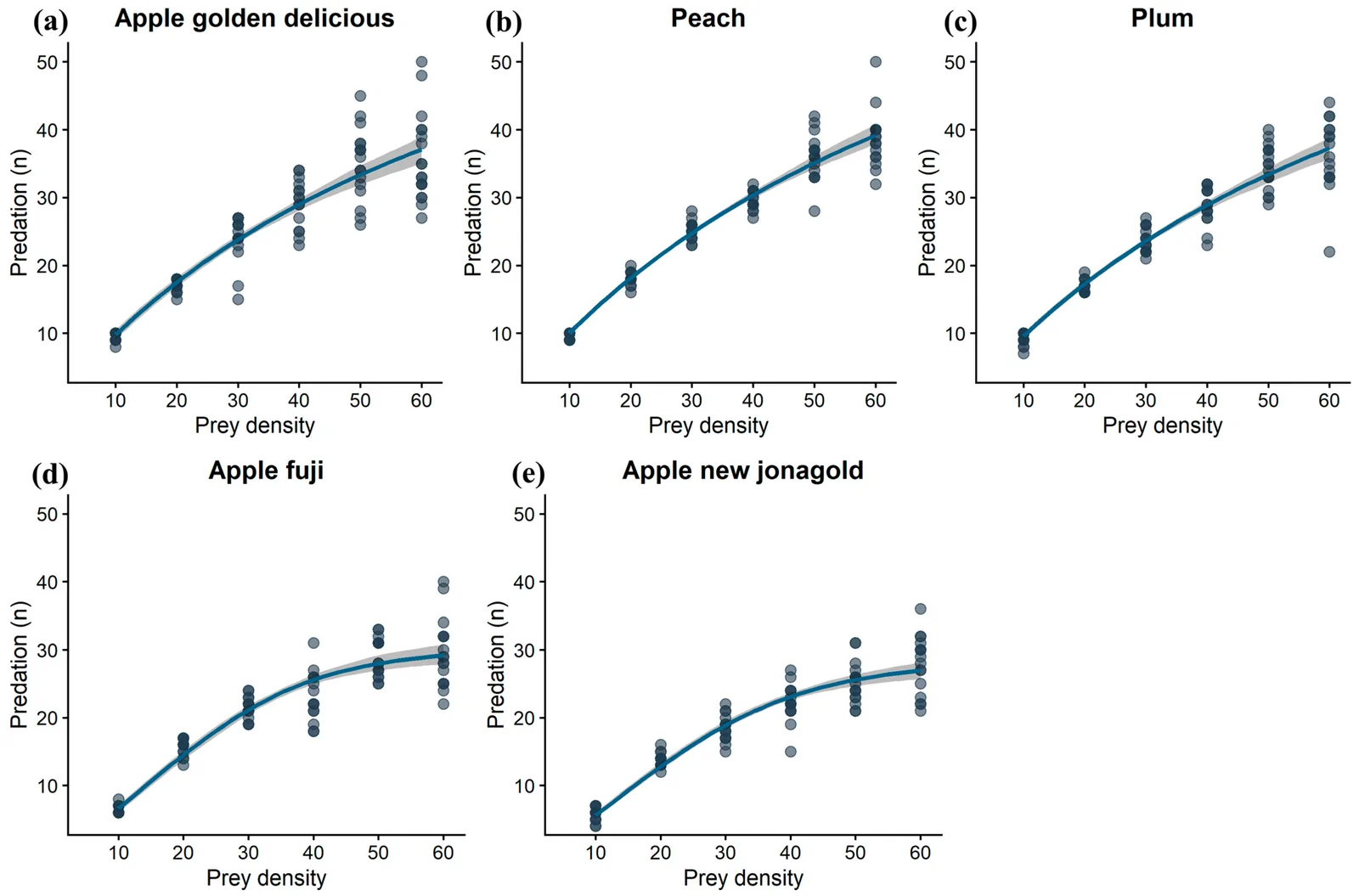
Functional response of *Scolothrips takahashii* fed on *Amphitetranychus viennensis* on different plant leaves. Shaded areas represent empirical approximations of 95% confidence intervals based on bootstrapped model fits. The circles show the predation of *Scolothrips takahashii* at different prey densities. (**a**) Apple Golden Delicious, (**b**) Peach, and (**c**) Plum matched the type II functional response model; (**d**) Apple Fuji and (**e**) Apple New Jonagold matched the type III functional response model.

The functional response of *S. takahashii* is influenced by the host plants of its prey, *A. viennensis*. The positive density coefficient in the logistic regression analysis indicates that female adults of *S. takahashii* feeding on *A. viennensis* eggs on Fuji and New Jonagold apples exhibit a type III functional response. In contrast, negative density coefficients indicate that female adults of *S. takahashii* feeding on *A. viennensis* eggs from Golden Delicious apples, peaches, and plums exhibited a type II functional response (Table 2, Figure 1).

The attack rate and handling time for type II functional response were not significantly affected by host plant, since the 95% confidence intervals for all evaluated cases overlapped (Table 3). When comparing the two prey sources exhibiting type III functional responses (Fuji and New Jonagold apples), the attack rate of *S. takahashii* on eggs on Fuji was significantly higher than that on New Jonagold (95% confidence intervals did not overlap), but there was no significant difference in handling time (Table 3).

Under all prey density conditions, there were significant differences in the egg consumption by *S. takahashii* on different host plants (Table 4). Overall, at each density level, the egg consumption on the three host plants (Golden Delicious apples, peaches, and plums) exhibiting a type II functional response was significantly higher than that on the two host plants (Fuji apples and New Jonagold apples) exhibiting a type III functional response. At the same time, the predation rate of *S. takahashii* was significantly influenced by density, host plant, and their interactions (Table S1). Furthermore, compared to Apple/Fuji, the predation rate of Apple/New Jonagold did not show a significant difference as density increased, but the predation rate of type II (Apple/Golden Delicious, Peach, Plum) declined more rapidly as density increased (Table S2).

## 4. Discussion

This study revealed that different Rosaceae host plants can significantly alter the functional response type of *S. takahashii* to *A. viennensis* eggs, shifting it from the common type II to type III. Our results clearly indicate that on two apple cultivars (Fuji and New Jonagold) with high trichome density, female adults of *S. takahashii* exhibited a type III functional response when feeding on *A. viennensis* eggs reared on the corresponding host plants, whereas on Golden Delicious apples, peaches, and plums with lower trichome density, they exhibited the classic type II functional response. This finding represents the first confirmation of a similar phenomenon in predatory thrips as natural enemies, following Merlin et al. (2022) [20], who identified host plant-induced type III functional responses in predatory mites. It underscores the central role of plant physical traits in three-trophic-level interactions [9].

The transformation of the functional response type of *S. takahashii* was closely associated with the density of trichomes on host plant leaves. The undersides of Fuji and New Jonagold apple leaves are densely covered with numerous, relatively long trichomes, which may physically impede the thrips’ foraging and predation behaviors. At low prey densities, this barrier effect may be particularly pronounced, causing thrips to spend more time moving and locating prey, thereby exhibiting a lower predation rate. This phenomenon is a typical characteristic of type III responses in low-density ranges [27,28]. As prey density increases, the probability of random encounters between predators and prey rises, partially overcoming the trichome barrier, and causing the predation rate to rise accordingly. However, the predation rate on leaves with low trichome density remains higher than that on leaves with high trichome density. This mechanism is similar to the findings of Barbosa et al. (2019) [13], who discovered that dense trichomes on tomato and melon leaves significantly reduced the predation of whitefly eggs by predatory mites. In contrast, our study goes a step further by demonstrating that this physical barrier effect is sufficiently strong to alter the functional response curve. Notably, on Golden Delicious apples, peaches, and plums with equally low trichome densities, thrips consistently exhibited a type II response, further confirming that high trichome density could be the key driver of the shift in functional response type.

In addition to physical barriers, a decline in the nutritional quality of prey may be another important mechanism leading to type III functional responses. A study by Merlin et al. (2022) provides direct evidence: when two-spotted spider mites, *Tetranychus urticae*, fed on low-quality host plants (cotton), the caloric content of their eggs decreased significantly, causing the predator, *Neoseiulus californicus*, to exhibit a type III functional response [20]. In our system, hawthorn spider mites may face greater feeding challenges when consuming apple leaves with high trichome density. For example, they may need to avoid trichomes or face stronger defensive responses from the plant. These challenges may lead to reduced nutrient accumulation in their bodies and, consequently, a lower nutritional value of their eggs [29]. Although this study did not directly measure the nutritional composition of the mite eggs, the significant prolongation of the predator’s handling time (*h*) can indirectly reflect a decline in prey quality. We found that in the type III response group (Fuji and New Jonagold), the thrips’ handling time (0.78–0.84 h) was significantly longer than in the type II response group (0.44–0.45 h). The prolonged handling time accounts not only for physical chewing and digestion but may also reflect the predator’s efforts to compensate for nutritional intake from low-quality prey [30,31]. We hypothesize that high trichome density may contribute to the shift in the functional response of *S. takahashii* toward type III through direct physical obstruction and a potential indirect reduction in prey quality. This hypothesis requires further analysis of the nutritional composition of the eggs to verify it.

Interestingly, Li et al. (2006) found that treatment with sublethal doses of abamectin and fenpropathrin could also shift the functional response of *S. takahashii* from type II to type III [22]. Based on the results of this study, we propose a new hypothesis: both chemical stress (pesticides) and physical/nutritional stress (unfavorable host plants) may induce predators to adopt similar adaptive predation strategies. When faced with stress, predators may reduce their search and attack activities in environments with low prey density (possibly due to poisoning, mobility constraints, or active avoidance of low-quality prey), resulting in abnormally low predation rates. Conversely, when prey density increases, their predatory drive and efficiency are fully stimulated [21]. This “stress-induced type III functional response” may represent a widespread yet previously overlooked ecological phenomenon. From the perspective of biological control, type III functional responses are generally considered more conducive to population system stability than type II responses, as they provide a “refuge” at low densities while exerting strong density-dependent control at high densities [6,7]. However, for pest management, the type III response implies that natural enemies have a weaker control effect during the early stages of a pest outbreak (low density), which may allow pests to more easily survive the low-density bottleneck and subsequently explode in numbers [32]. At the same time, this study found that the predation rate associated with type II functional responses remains relatively high at low densities, but as prey density continues to increase, it declines more rapidly than the predation rate in type III responses. These findings suggest that if the growth rate of the prey population exceeds the predation rate, the growth of the prey population cannot be suppressed. Therefore, in the practical application of using *S. takahashii* to control hawthorn spider mites, orchards growing high-trichome apple cultivars such as Fuji and New Jonagold need to carefully evaluate the effectiveness of early natural enemy releases and consider supplementing with other biological control measures when mite densities are low. However, in orchards growing low-trichome crops such as Golden Delicious apple, peach, and plum, the natural enemy should be released as early as possible during outbreaks to achieve effective biological control.

Furthermore, our results reveal that significant differences exist among different cultivars of the same species. Although both Fuji and New Jonagold induced a type III response, there was a significant difference in the attack rate coefficient (*b*), indicating that the trichome traits of different cultivars (which may include subtle differences in morphology and secretions, etc.) exert varying degrees of influence on natural enemies. This suggests that when conducting pest-resistant variety breeding or screening for natural enemy-friendly cultivars, we should not rely solely on simple criteria such as hairy or hairless, but rather conduct more detailed quantitative assessments.

## 5. Conclusions

In summary, this study demonstrates that host plants, particularly their leaf trichome characteristics, can profoundly influence the functional response type of *S. takahashii*. The findings represent an important contribution to the theory of predator–prey dynamics and provide practical guidance for the biological control of orchard pests. Future research should further quantify the direct nutritional composition of spider mite eggs on different host plants (e.g., protein, lipid, and carbohydrate content). Additionally, it should conduct functional response validation under semi-field or field conditions to comprehensively assess the practical impact of plant traits on the efficacy of natural enemy pest control. Exploring whether the plant-mediated type III functional response can be altered through transgenerational acclimation of natural enemies is also a topic worthy of further investigation.

## Figures and Tables

**Table 1:** Number and length (±SE) of trichomes on leaves of five plants.

Plant/Cultivar	Density (per cm^2^)	Length (mm)
Apple/Golden Delicious	14.40 ± 1.92 c	1.247 ± 0.103 b
Apple/Fuji	36.50 ± 2.79 b	1.799 ± 0.142 ab
Apple/New Jonagold	48.80 ± 3.89 a	2.296 ± 0.228 a
Peach	8.32 ± 1.16 c	1.148 ± 0.156 b
Plum	10.56 ± 1.28 c	1.206 ± 0.138 b

Different letters within a column indicate significant differences between the treatments (*p* < 0.05), based on the result of Tukey’s HSD.

**Table 2:** Maximum likelihood estimates from logistic regression of the proportion of *Amphitetranychus viennensis* eaten by *Scolothrips takahashii* on initial prey density on different host plants.

Plant/Cultivar	Type	Coefficient	Estimate	SE	z-Value	Pr (z)
Apple/Golden Delicious	II	Intercept Density Density^2 Density^3	4.710 −0.2344 0.005279 −0.00004285	0.7688 0.068 0.001856 0.00001573	6.143 −3.447 2.844 −2.724	<0.0001 <0.0001 0.004457 0.006454
Apple/Fuji	III	Intercept Density Density^2 Density^3	−0.08701 0.1239 −0.004036 0.00003324	0.464 0.04645 0.001364 0.0000121	−0.1887 2.668 −2.96 2.746	0.85125 0.00763 0.00308 0.00603
Apple/New Jonagold	III	Intercept Density Density^2 Density^3	−3.533 0.1085 −0.003502 0.00002924	0.4512 0.045 0.001323 0.00001177	−0.783 2.411 −2.647 2.483	0.43364 0.0159 0.00811 0.01303
Peach	II	Intercept Density Density^2 Density^3	5.596 −0.2444 0.004626 −0.00003234	1.016 0.08531 0.002245 0.00001857	5.509 −2.864 2.06 −1.741	<0.0001 0.00418 0.0394 0.08167
Plum	II	Intercept Density Density^2 Density^3	3.917 −1.654 0.003364 −0.00002623	0.7289 0.06512 0.001791 0.00001528	5.373 −2.54 1.878 −1.716	<0.0001 0.0111 0.0604 0.0861

**Table 3:** Attack rate (*a*; *b*) and handling time (*h*) estimates (±SE) of *Scolothrips takahashii* fed on *Amphitetranychus viennensis* on different host plants.

Plant/Cultivar	Type	*a*; *b* * (prey.h^−1^)	95% C.I.	*h* (h)	95% C.I.
Apple/Golden Delicious	II	0.126 ± 0.00089	(0.1097–0.1445)	0.4524 ± 0.02476	(0.4023–0.4996)
Apple/Fuji	III	0.01135 ± 0.0013	(**0.0091**–0.0143)	0.7841 ± 0.0223	(0.7404–0.8281)
Apple/New Jonagold	III	0.0073 ± 0.00084	(0.00579–**0.00903**)	0.8358 ± 0.0282	(0.7769–0.8877)
Peach	II	0.1489 ± 0.0111	(0.1287–0.1724)	0.4505 ± 0.0235	(0.4028–0.4953)
Plum	II	0.1179 ± 0.0085	(0.1024–0.1358)	0.4350 ± 0.0268	(0.3805–0.4858)

* *a*: attack rate for type II functional response; *b*: attack rate for type III functional response; Bold numbers in the table indicates a significant difference between the two groups.

**Table 4:** Consumption (mean ± SE) of *Amphitetranychus viennensis* by *Scolothrips takahashii* at various prey densities on five host plants.

Density of Prey (Individuals/Dish)	Apple/ Golden Delicious	Apple/ Fuji	Apple/ New Jonagold	Peach	Plum
10	9.47 ± 0.14 a	6.56 ± 0.15 b	5.82 ± 0.23 b	9.71 ± 0.11 a	9.29 ± 0.22 a
20	17.00 ± 0.22 ab	15.67 ± 0.32 bc	13.80 ± 0.26 c	18.13 ± 0.26 a	16.93 ± 0.25 ab
30	23.80 ± 0.91 ab	21.47 ± 0.43 bc	18.47 ± 0.51 c	25.19 ± 0.34 a	23.75 ± 0.45 ab
40	29.13 ± 0.93 a	23.20 ± 0.95 b	22.20 ± 0.74 b	29.60 ± 0.36 a	28.80 ± 0.73 a
50	35.06 ± 1.27 a	28.94 ± 0.68 b	25.18 ± 0.72 c	36.06 ± 0.82 a	34.53 ± 0.80 a
60	35.83 ± 1.50 a	29.47 ± 1.21 b	27.81 ± 1.07 b	38.63 ± 1.04 a	36.38 ± 1.34 a

Means ± SE within a row followed by different letters are significantly different (density 10–30: Kruskal–Wallis/Dunn’s test; density 40–60: Tukey’s HSD; *p* < 0.05).

## Data Availability

The data presented in this study are available in the article.

## Supplementary Materials

The following supporting information can be downloaded at https://www.mdpi.com/article/10.3390/insects17080840/s1. Table S1. Generalized linear models were used to analyze the effects of density, host plants, and their interactions on predation rates. The bold value is the significant value. Table S2. Parameter estimates for the binomial GLM model.
