# Supplementary material for: Host Plant Effects on the Functional Response of Scolothrips takahashii to Amphitetranychus viennensis
Source: Insects. 2026 Aug 13;17(8):840. doi: 10.3390/insects17080840 (PMC13513148; doi:10.3390/insects17080840)
Supplement: Supplementary file 1 [file insects-17-00840-s001.zip › insects-4432693-supplementary.pdf]

Table S1. Generalized linear models were used to analyze the effects of density, host plants, and their interactions on predation rates. The bold value is the significant value.

	Chisq	Df	*P*
Density	601.47	1	<0.0001
Plant/Variety	503.94	4	<0.0001
Density: Plant/Variety	42.99	4	<0.0001

Table S2. Parameter estimates for the binomial GLM model.

	Estimate	Std	z	*Pr(>|z|)*
Density	-0.0238	0.00246	-9.69	<0.0001
Apple/Golden Delicious	1.09	0.183	5.95	<0.0001
Apple/New Jonagold	-0.510	0.161	-3.17	0.001515
Peach	1.43	0.195	7.37	<0.0001
Plum	0.943	0.182	5.17	<0.0001
Density: Apple/Golden Delicious	-0.0115	0.00377	-3.05	0.002269
Density: Apple/New Jonagold	0.00615	0.00343	1.79	0.073048
Density: Peach	-0.0151	0.00400	-3.78	0.000157
Density: Plum	-0.00881	0.00380	-2.32	0.020328

Note: Apple/Fuji is the reference level.
